# Phosphorus deficiency induces sexual reproduction in the dinoflagellate *Prorocentrum cordatum*

**DOI:** 10.1038/s41598-023-41339-3

**Published:** 2023-08-30

**Authors:** Vera Kalinina, Mariia Berdieva, Nikolay Aksenov, Sergei Skarlato

**Affiliations:** 1 Laboratory of Cytology of Unicellular Organisms, Institute of Cytology of the Russian Academy of Sciences, St.-Petersburg, 194064 Russia; 2 Laboratory of Intracellular Membrane Dynamics, Institute of Cytology of the Russian Academy of Sciences, St. Petersburg, 194064 Russia

**Keywords:** Circadian rhythms, Cell biology, Cell division, Meiosis, Mitosis, Microbiology, Water microbiology

## Abstract

Nitrogen (N) and phosphorus (P) are essential elements whose availability promotes successful growth of phytoplankton and governs aquatic primary productivity. In this study, we investigated the effect of N and/or P deficiency on the sexual reproduction of *Prorocentrum cordatum*, the dinoflagellate with the haplontic life cycle which causes harmful algal blooms worldwide. In *P. cordatum* cultures, N and the combined N and P deficiency led to the arrest of the cell cycle in the G_0_/G_1_ phases and attenuation of cell culture growth. We observed, that P, but not N deficiency triggered the transition in the life cycle of *P. cordatum* from vegetative to the sexual stage. This resulted in a sharp increase in percentage of cells with relative nuclear DNA content 2C (zygotes) and the appearance of cells with relative nuclear DNA content 4C (dividing zygotes). Subsequent supplementation with phosphate stimulated meiosis and led to a noticeable increase in the 4C cell number (dividing zygotes). Additionally, we performed transcriptomic data analysis and identified putative phosphate transporters and enzymes involved in the phosphate uptake and regulation of its metabolism by *P. cordatum*. These include high- and low-affinity inorganic phosphate transporters, atypical alkaline phosphatase, purple acid phosphatases and SPX domain-containing proteins.

## Introduction

Dinoflagellates are one of the most important groups of protists contributing to marine primary production, transfers of matter and energy within the aquatic food chains, and formation of harmful algal blooms worldwide, also called “red tides”^1, 2^. These microalgae have a complex life cycle with altering of vegetative and transient sexual stages. With the only known exception of *Noctiluca*^3^, typical dinoflagellates have a haplontic life cycle. Under certain environmental conditions, vegetative cells differentiate into gametes that fuse and form a diploid zygote. The latter either forms a sexual resting cyst or undergoes two successive meiotic divisions without long-term encystment. During germination, sexual resting cysts go through meiosis^4–6^.

The search for factors regulating the transition from the vegetative to the sexual stage of the life cycle has been the subject of research for several decades. In some species, this transition can be spontaneous^3, 7^. However, like in many other protists, sex in dinoflagellates is regarded as a survival strategy in adverse environmental conditions, such as nutrient deprivation, unfavorable temperature regime, salinity stress, grazing, or parasite infection^8–11^. Interactions between the factors inducing sexuality may be complex. For example, in *Alexandrium minutum*, the combined effect of three factors, such as P deficiency, low salinity, and high temperature significantly increase the yield of the planozygotes and sexual resting cysts in comparison with the effect of the only P and/or N deficiency^9^. The presence of an appropriate mating partner is also important for successful sexual reproduction in heterothallic species^12^. For many dinoflagellates, the sexual process was described in late exponential or stationary cultures, that are characterized by high cell concentration and depletion of nutrient sources^13–15^. In heterotrophic species, such as *Polykrikos kofoidii*, the absence of prey can also induce gamete formation^7^. Indeed, nutrient availability is likely to play a key role in the regulation of life cycles in this group of protists. In cultures of most dinoflagellates, sexuality can be induced by lowering or eliminating the N and/or P or by changing their ratio in the culture medium^8, 16^.

*Prorocentrum cordatum* (currently accepted taxonomically synonym of *Prorocentrum minimum*) is a neritic planktonic dinoflagellate and the causative agent of harmful algal blooms throughout the world^17–21^. The life cycle of *P. cordatum* has been recently described^22^. Different sexual stages, such as fusing gametes, planozygotes, and zygotes dividing by meiosis were observed in the aging culture, which was growing without adding fresh medium and nutrients for more than 3 weeks. However, the specific factors that trigger a transition from vegetative reproduction to sex have not been reported so far. We suggested that N and/or P deficiency can trigger sexual reproduction in *P. cordatum*.

Since the transition from the vegetative stage of the life cycle to the sexual stage is primarily regulated by nutrient scarcity, molecular transporters and enzymes involved in N and P uptake and the first steps of their metabolism can participate in signal transmission from the environment and the regulation of the life cycle. The transporters and enzymes involved in N acquisition have been listed and characterized in *P. cordatum* in previous works. These are DUR3 and major intrinsic protein (MIP), involved in urea transport, nitrate transporter NRT2, urease, assimilatory nitrate reductase NR and nitrite reductase NIR^23–26^. However, little is known about the molecular machinery involved in P acquisition. The only well-characterized enzyme is alkaline phosphatase (AP), which releases inorganic phosphate (Pi) from various types of phosphoester molecules. In dinoflagellates, AP is a key cell surface hydrolase, which allows the utilization of dissolved organic phosphorus (DOP) when Pi is scarce. Sequence comparisons and phylogenetic analyses of dinoflagellate APs elucidated that they comprise an atypical type of AP – PhoA^aty^, which is similar to those reported in proteobacteria, cyanobacteria, green algae, haptophytes, and stramenopiles^27^. In *P. cordatum*, the expression and activity of this enzyme increase in a P-limited environment^28, 29^. Interestingly, in some dinoflagellates, for instance in *Karenia brevis* and *Alexandrium catenella* functional activity of AP but not expression increases under P-starvation^30–32^.

Eukaryotic microorganisms possess low-affinity Pi transporters that function at high Pi concentrations and high-affinity Pi transporters that are up-regulated under Pi deficiency. The low-affinity Pi transporters subdivide into Pi transporters IPT and the sodium- or sulfate-dependent Pi transporters SPT. Lin and coauthors^33^ systematized the knowledge about P transporters in eukaryotic phytoplankton and performed screening of several dinoflagellate transcriptomes to find sequences of putative Pi transporters. The homologs of both high-affinity and low-affinity Pi transporters including SPT were found. However, none of these transcriptomes contain a fool set of these transporters^33^.

In this study, we basically focused on two fundamental questions: does nutrient deficiency regulate the life cycle of *P. cordatum* and which molecular machinery can be involved in this process? We investigated how the absence of N and/or P affects culture growth and the cell and life cycles. Our findings revealed that P-deficiency triggers a transition from the vegetative stage to the sexual stage. To shed further light on these mechanisms, we screened publicly available *P. cordatum* transcriptomes, listed key genes involved in P uptake and suggested that some of them might participate in the regulation of the life cycle.

## Results

### Nitrate and phosphate uptake during culture growth

**Figure 1:**
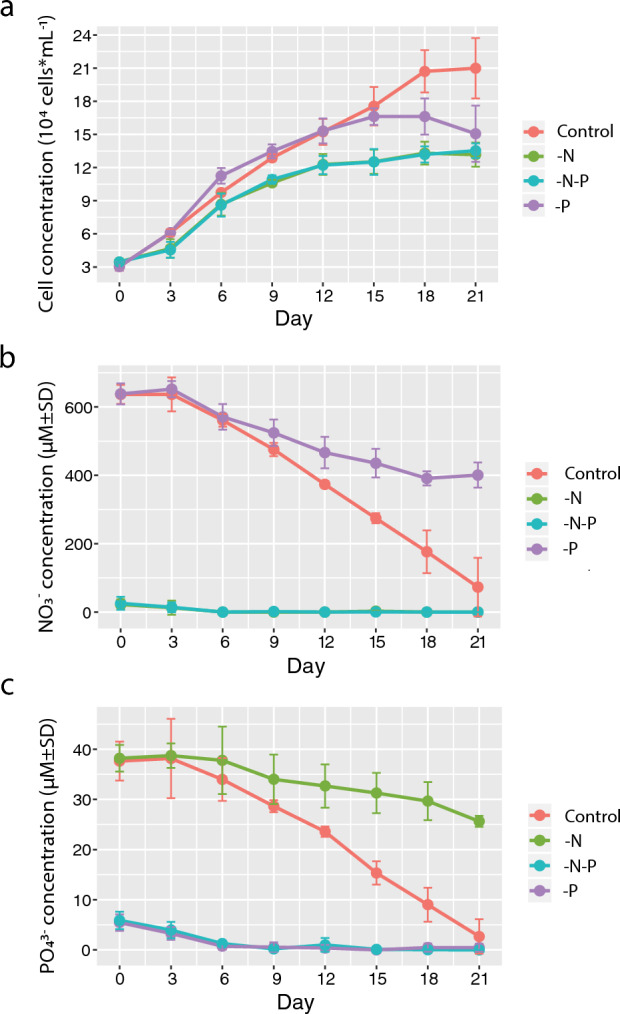
Growth curves (**a**), nitrate, and phosphate concentrations (**b**,**c**) in the *P. cordatum* cultures under the control, N-, P-, and N-P-limited conditions over the 21-day experimental period. Data shown are means ± standard errors of the mean (**a**) and standard deviations (**b**,**c**) for the triplicated experiments.

Control and P-deficient cultures exhibited similar growth rates up to day 15 (Fig. 1a, Table 1). The P-deficient culture reached a plateau in 15 days with maximum biomass 1.66 × 10^5^ cell mL^–1^, while control cells continued to divide until day 18 and reached a cell density 2.09 × 10^5^ cell mL^–1^ by the end of the experiment. The growth of N- and N-P-deficient cultures was impaired from the beginning and the difference became considerable in 9 days when the cell concentrations in N- and N-P-deprived cultures reached 1.06 × 10^5^ and 1.09 × 10^5^ cell mL^–1^ respectively, and in the control and P-deficient cultures were 1.29 × 10^5^ and 1.35 × 10^5^ cell mL^–1^ respectively. The maximum biomass in N- and N-P-deprived cultures at day 21 was only 1.35 × 10^5^ cell mL^–1^ (Fig. 1a).

**Table 1:** The specific growth rate in control, P-, N- and N-P-deficient cultures during 21 days of the experiment.

Day of experiment	Control		P-deficient		N-deficient		N-P-deficient	
	Growth rate	S-phase	Growth rate	S-phase	Growth rate	S-phase	Growth rate	S-phase
3	0.23	–	0.23	–	0.10	–	0.09	–
6	0.16	0.26	0.20	0.26	0.21	0.18	0.21	0.18
9	0.09	0.17	0.06	0.07	0.07	0.07	0.08	0.08
12	0.06	0.15	0.04	0.08	0.05	0.03	0.04	0.04
15	0.05	0.11	0.03	0.13	0.01	0.02	0.01	0.05
18	0.06	0.08	0.00	0.03	0.02	0.01	0.02	0.01
21	0.00	0.08	− 0.03	0.01	0.00	0.01	0.01	0.01

Columns for S-phases gives the proportion of cells in S-phase at 5:00.

In the control, nitrate and phosphate concentrations gradually decreased from 630 to 72 µM and from 37 to 2 µM respectively by the end of the experiment (Fig. 1b,c). In N- and N-P-deficient cultures, nitrate was undetectable starting from day 6. Nitrate uptake was significantly impaired in the P-deficient culture, compared to the control (Fig. 1b). In P- and N-P-deprived cultures, phosphate concentration in the growth medium dropped below the detection limit in 9 days. Phosphate uptake was significantly reduced in the N-deprived culture (Fig. 1c).

### The role of N and P deficiency in the regulation of cell and life cycle

**Figure 2:**
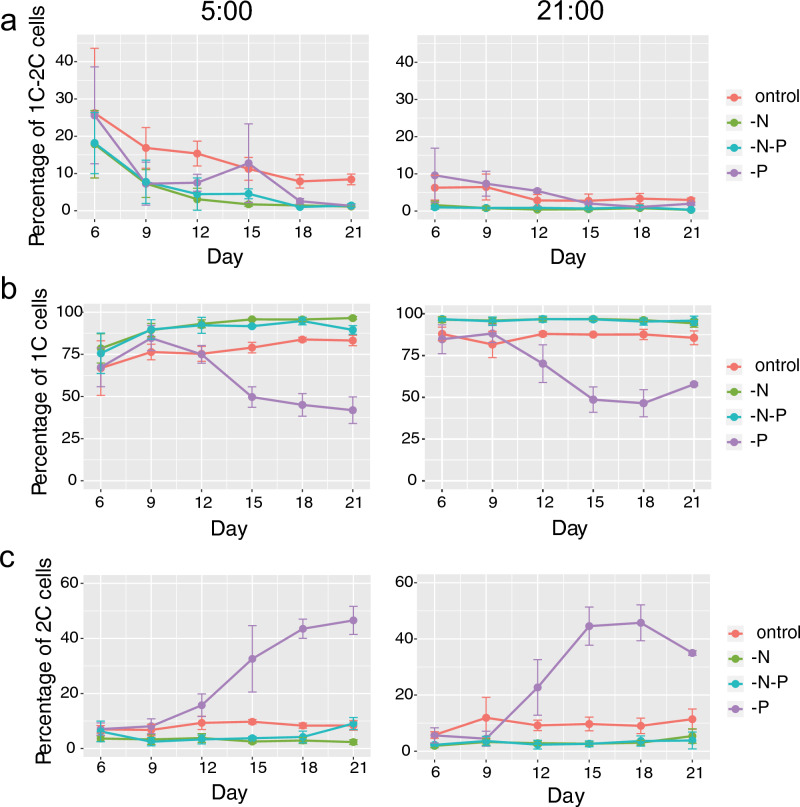
Percentage of cells with relative nuclear DNA content 1C, 1C → 2C and 2C during the 21-day experimental period at 5:00, and 21:00. (**a**) Percentage of cells with relative nuclear DNA content 1C → 2C (S-phase of the cell cycle). (**b**) Percentage of cells with relative nuclear DNA content 1C (G_0_/G_1_-phase). (**c**) Percentage of cells with relative nuclear DNA content 2C (G_2_/M-phase and zygotes). Shown are means ± standard errors of the mean from the triplicated experiments.

The proportion of cells with relative DNA content 1C → 2C (S-phase) at 5:00 (maximum cell number expected during S phase) correlated with the calculated specific growth rate in all cultures (Table 1). In control, the number of cells in the 1C → 2C stage gradually decreased from 26% at day 6 to 8% by the end of the 3-weeks experiment. In the P-deficient culture, the percentage of 1C → 2C cells decreased at the same rate as in the control up to day 15, then the number of cells in the S-phase became close to zero. In N- and N-P-deficient cultures, the percentage of cells in the S-phase dropped below 5% by day 12 (Fig. 2a). At 21:00 (minimum cell number expected during S phase), the percentage of cells in the S-phase was low in all experiments, and the changes in this proportion during the experiment were negligible.

In control at 5:00, N-deficient, and N-P-deficient cultures, the fraction of cells in the 1C stage slightly increased from 6 to 9 days (Fig. 2b) in accordance with the decrease in the proportion of 1C → 2C cells (Fig. 2a). The number of 2C cells did not significantly change and was about 10% in control and 3–5% in N-deficient and N-P-deficient cultures during the whole experiment (Fig. 2c).

In P-deficient culture, the percentage of cells with relative DNA content 2C clearly increased from 5–8% on day 9 up to 22–32% on day 12 and reached ~ 45% plateau in 15 days (Fig. 2c). The fraction of 1C cells decreased in line with the rise of the proportion of the 2C stage (Fig. 2b). Surprisingly, a small fraction of cells with relative DNA content 2C → 4C and 4C appeared starting from day 12 and reached 3% and 7% respectively by day 21 (Suppl. Figs. 1, 2).

**Figure 3:**
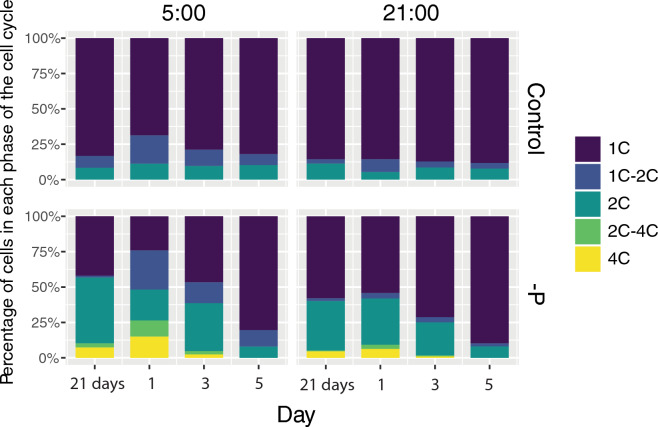
Percentage of cells in the different phases of the cell cycle after 21 days of P-deficiency cultures and 1, 3 and 5 days after adding fresh medium.

Suggesting that the cells with relative nuclear DNA content 2C and 4C are the sexual stages, we assumed the addition of nutrients can stimulate meiosis and return to the initial vegetative state. After adding the f/2 medium containing the required concentration of nitrate and phosphate, changes in the ratio of cells with different DNA content were most pronounced at 5:00. In the P-deprived culture, the proportion of the 4C stage at 5:00 more than doubled in 24 h period and reached 15%. Moreover, the proportion of 1C → 2C cells increased sharply from 1.3 to 27% at day 1. In three days, the fractions of 4C and 2C → 4C stages significantly decreased and vanished by day 5, when the proportion of cell cycle stages became indistinguishable from the control. After adding the fresh medium to the control culture, the proportion of 1C → 2C cells also doubled in 24 h but returned to initial values in 3 days. Similar, but less pronounced trends were observed at 21:00 in both control and P-deprived cultures (Fig. 3).

### Proteins involved in phosphate uptake revealed by transcriptome analysis

Given that phosphorus deficiency causes a shift in the life cycle progress in *P. cordatum*, we focused on searching for proteins responsible for phosphate uptake in publicly available transcriptomes. Annotated sequences of budding yeasts *Saccharomyces cerevisiae,* higher plant *Arabidopsis thaliana,* and haptophyte *Emiliania huxleyi* were used as queries. The full list of queries, their functions, and sequences found in *P. cordatum* is shown in Table 2.

**Table 2:** List of queries and the sequences of putative proteins involved in phosphate acquisition, transport, and metabolism in the transcriptomes of *P. cordatum* strains CCAP2233 and CCAP1329.

Query				*P. cordatum* homologs			
GenBank or RefSeq ID	Title	Function	Organism	Strain CCAP2233	E-value	Strain CCAP1329	E-value
QHB10616.1	PHO84	High-affinity inorganic phosphate transporter	*Saccharomyces cerevisiae*	CAMPEP_0191146536^**a**^ CAMPEP_0191095568^**b**^ CAMPEP_0191040874 CAMPEP_0191156728^**c**^	5e-47 1e-42 1e-36 4e-34	CAMPEP_0190942338^**a**^ CAMPEP_0190908170^**b**^ CAMPEP_0190869834 CAMPEP_0191021556^**c**^	5e-47 2e-44 9e-37 9e-27
KAF4005909.1	PHO91	SPX-containing low-affinity phosphate transporter	*Saccharomyces cerevisiae*	CAMPEP_0191068652^**d**^ CAMPEP_0191065880^**e**^ CAMPEP_0191121806 CAMPEP_0191121060	4e-28 6e-26 1e-16 1e-16	CAMPEP_0190869750^**d**^ CAMPEP_0190865364^**e**^ CAMPEP_0190956556	3e-28 5e-26 3e-17
QHB09608.1	VTC4	Vacuolar transporter chaperone complex subunit 4	*Saccharomyces cerevisiae*	CAMPEP_0191116404^**f**^ CAMPEP_0191105028^**g**^	5e-60 8e-12	CAMPEP_0191031170^**f**^ CAMPEP_0190931754^**g**^	3e-60 8e-12
KAG2518769.1	IPP1	Inorganic pyrophosphatase	*Saccharomyces cerevisiae*	CAMPEP_0191103562^**h**^ CAMPEP_0191105952 CAMPEP_0191105238 CAMPEP_0191105952 CAMPEP_0191184226 CAMPEP_0191183006	3e-60 8e-25 5e-20 1e-18 5e-16	CAMPEP_0190910846^**h**^ CAMPEP_0190881396 CAMPEP_0191022770	3e-60 1e-55 4e-21
OAP03352.1	PHO1	Phosphate transporter	*Arabidopsis thaliana*	CAMPEP_0191052604	1e-25	–	–
AEE74502.1	PAP15	purple acid phosphatase	*Arabidopsis thaliana*	CAMPEP_0191092762 CAMPEP_0191040114^**i**^ CAMPEP_0191122392 CAMPEP_0191107316 CAMPEP_0191157012 CAMPEP_0191182548 CAMPEP_0191039590^**j**^ CAMPEP_0191055416	4e-72 9e-61 4e-49 8e-49 1e-48 1e-34 2e-21 8e-20	CAMPEP_0190872632 CAMPEP_0190918190^**i**^ CAMPEP_0191014798 CAMPEP_0191009958 CAMPEP_0190902674 CAMPEP_0190937684^**j**^	2e-63 8e-61 1e-49 3e-49 7e-49 2e-21
AWI47784.1	PhoA1	Alkaline phosphatase	*Emiliania huxleyi*	CAMPEP_0191092442^**k**^	0	CAMPEP_0190946092^**k**^	0
NP_197515.1	SPX1	SPX domain-containing protein 1	*Arabidopsis thaliana*	CAMPEP_0191187852	6e-005	–	–

Identical sequences in two transcriptomes are marked with the same superscript letters.

The *pho84* gene encodes a high-affinity H^+^-phosphate symporter in *S. cerevisiae* which belongs to the major facilitator superfamily (MFS). We found 16 sequences of 12 homologs in two *P. cordatum* transcriptomes producing significant alignments with an e-value less than e^–10^. Five homologs (8 sequences in two transcriptomes) contained glycine-rich phosphate binding motif GXGXGG shared by proton-coupled phosphate transporters in plants, fungi, bacteria, and mammals (Suppl. Fig. 3)^34^. These sequences were included in the final list of homologs (Table 2). Also, the sequences were analyzed for the presence of conservative motifs of proton-coupled transporters of plant and fungal origin generated by the motif-building program MEME^35^. Three out of five motifs were found in *P. cordatum* homologs (Suppl. Fig. 3). In addition, sites corresponding to *S. cerevisiae* Arg168, Asp358, and Lys492 shown to be critical for the transport function in PHO84 are present in *P. cordatum* homologs. We also retrieved five low-affinity phosphate transporters, homologous to yeast’s PHO90, PHO91, and PHO87. It should be noticed that in yeasts these proteins contain the SPX domain, but it is absent in *P. cordatum* homologs.

Alkaline phosphatase (AP) is a hydrolase, which releases Pi from organic molecules. For the search of the APs, we used the sequence of haptophyte *E. huxleyi* as a query, which represents atypical AP PhoA^aty^. We found singular sequences with strong homology (*E*-value = 0) in each present transcriptome of *P. cordatum*. Purple acid phosphatases (PAPs)—the members of the metallo-phosphoesterase family—can also be important for Pi liberation from the phosphate monoesters. Using the amino acid sequence of *A. thaliana* PAP15 (GenBank ID: AEE74502.1) as a query against two *P. cordatum* transcriptomes, 12 putative PAP homologs were retrieved (Table 2). We performed multiple sequence alignment of all *P. cordatum* PAP homologs with PAP11 and PAP12 of *A. thaliana* using the ClustalO algorithm and found that all 5 conservative motifs mentioned by Schenk and coauthors^36^ are present in *P. cordatum* PAPs (Suppl. Fig. 4).

We also revealed five SPX domain-containing sequences in two *P. cordatum* transcriptomes. When we used *A. thaliana* SPX domain-containing protein 1 as a query, the sequence homology was too low (*E*-value less than e^–10^). Therefore, we re-verified the presence of the SPX domain using the NCBI Conserved Domain Search database and PROSITE. Among retrieved sequences, there is one containing the SPX domain as the sole functional domain. Two SPX-containing homologs (four sequences) possess a vacuolar transporter chaperone (VTC) domain. One of them (CAMPEP_0191116404 and CAMPEP_0191031170) exhibits strong homology (*E*-value = 5e^–60^) to an *S. cerevisiae* catalytic subunit VTC4 of the VTC complex. In addition, we found one potential member of the *PHO1* gene family (CAMPEP_0191052604), which contains both SPX and EXS domains (Table 2). In *A. thaliana*, the PHO1 protein transfers Pi from the epidermal and cortical cells to the root xylem vessels.

## Discussion

**Figure 4:**
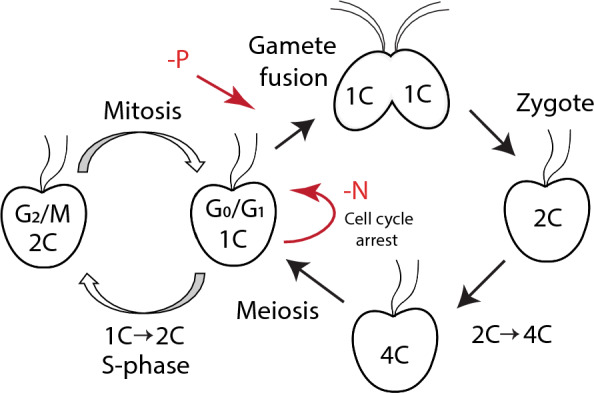
Schematic representation of the changes in the intracellular DNA content in the life cycle of the dinoflagellate *P. cordatum*.

According to its physiological demand, dinoflagellate blooms start and develop in favorable conditions, when nutrients are abundant. At this period of time, dinoflagellates reproduce vegetatively. During the peak bloom, when nutrient sources are depleted but the algal concentration is high, some cells turn to gamete formation and produce sexual resting cysts that sink to the bottom, and the bloom terminates in this way^5^. Recent studies revealed that meiosis genes are also expressed during bloom prolongation, suggesting that dinoflagellates use meiosis not only for encystment but also for cell proliferation^37^. Nevertheless, nutrient deficiency, especially N and P limitations, are considered to be the most effective factors to induce sexual reproduction in dinoflagellates^8, 16^. In this study, we demonstrated the role of N and P limitation in cell and life cycle changes in *P. cordatum* and summarized it in Fig. 4.

Both the quantity and quality of nutrients affect the dinoflagellate cell cycle and consequently the growth rate of these protists. The light–dark cycle has a strong control over the phases of the cell cycle in photosynthetic dinoflagellates. The timing of the S-phase and mitosis are not altered by the manipulation of nutrients, but N and P deficiency in the culture medium can result in the prolongation of the S-phase in *P. cordatum* cells^38, 39^. The decline of *P. cordatum* proliferation in nutrient-limited culture has also been shown in earlier studies^40, 41^. In the current work, growth was most strongly affected by N starvation, comparing to P deficiency. The number of cells entering the S-phase decreased along with nutrient depletion, which correlates well with the reduced growth rate. A deficiency in one nutrient can have a detrimental impact on the uptake of another. Our results are in line with the previous observations of Li and coauthors^40^ since we showed that P limitation negatively affected the N uptake and assimilation and alternatively, low N:P ratio led to N limitation of P uptake in *P. cordatum*.

Several studies investigated the physiological responses of *P. cordatum* to changing N:P ratio and N- and P-deficiency. The lack of N and P leads to an increase in intracellular C:N ratio, which is more pronounced in N-deprived cultures^24, 40, 41^, which appears to be associated with the accumulation of neutral lipids and starch in the cytoplasm, as it was shown in the relative species of *Prorocentrum lima*^42^. Under P limitation, photosynthesis efficiency declines^41^. Also, P but not N deficiency stimulates *P. cordatum* to get the missing nutrients from organic sources, inducing phagocytosis and increasing alkaline phosphatase expression and activity^43, 44^. However, none of these studies paid attention to life cycle changes. This could be attributed to the fact that gametes possess the same morphology as vegetative cells and can only be distinguished through “mating” behavior—pairing and swirling around each other. At the light-optical level fusing gametes resemble vegetative cells undergoing mitotic division. Moreover, *P. cordatum* does not produce sexual resting cysts, which serve as a pivotal marker of sex in cyst-forming species^22^. Deterioration of culture growth and certain physiological shifts, such as a decrease in photosynthesis efficiency caused by nutrient limitation can be associated not only with the arrest of the cell cycle in the G_0_/G_1_ phase; it could be attributed to the differentiation of some cells into gametes and subsequent zygote formation. Thus, it is reasonable to observe changes in relative intracellular DNA content in the cells in the experiments with nutrient deprivation.

Flow cytometry analysis of P-starved *P. cordatum* culture revealed a significant increase in the cell fraction with relative nuclear DNA content 2C on day 15 of the experiment. This phenomenon can be explained by a transition of the life cycle into the sexual phase, gamete production, and the appearance of 2C zygotes. This hypothesis is confirmed by the simultaneous appearance of 4C cell fraction, suggesting zygotes undergoing meiosis. Similar results were obtained in *A. minutum* cross cultures, where in a P-limited medium significant fraction of 4C cells appeared indicating sexual reproduction. At the same time, the number of sexual resting cysts in such cultures increased^45^. Moreover, 2C → 4C and 4C cells were found during blooms of *Prorocentrum shikokuense* and *Karenia mikimotoi*. Metatranscriptome profiling confirmed sexual reproduction by showing overexpression of meiosis genes in these dinoflagellates^37^.

With the development of sequencing technologies and their cost reduction, a considerable number of studies focused on transcription regulation under N or P deficiency in dinoflagellates. According to these studies, the genes involved in the sexual process did not appear to be overexpressed. However, it should be noticed that many dinoflagellates, for example, *A. minutum,* exhibit complex heterothallic mating types and need an appropriate genetically divergent sexual partner^46^. In this species, the sexual process can be induced by lowering N and/or P concentrations and enhanced by salinity decrease and temperature increase^9^. Transcriptomic analysis of N and P starved *A. minutum* culture revealed a long list of genes involved in sex determination, sex differentiation, and mating that were differentially expressed after 6 h of starvation. However, most of these unigenes returned to the normal expression level after 72 h of nutrient deficiency exposure^47^. This study suggests that N and P limitation does induce sexual reproduction in *A. minutum*, but the absence of appropriate mating partners would suppress this process. Another example is the toxic bloom-forming dinoflagellate *K. brevis,* which is also heterothallic*.* It was shown that a lack of N induces sexual reproduction and resting cyst formation in this protist^48, 49^. Microarray analysis of transcriptomic response revealed that neither N deficiency nor subsequent N addition induced expression of meiosis genes^31^*.* This result can be explained by the fact that the study was performed on a batch culture without sexual crosses.

Change from a depleted medium to a fresh one rich in nutrients leads to the germination of sexual cysts and meiosis in cyst-forming dinoflagellates^37^. In our experiments, the addition of fresh f/2 medium into the starving culture increased the proportion of 4C cells, that appear to be zygotes at the replication stage, in 24 h suggesting induction of meiotic divisions. This cell fraction decreased and disappeared in 5 days after nutrient supply, suggesting that all zygotes divided and the cells returned to the haploid state.

Many questions remain regarding how *P. cordatum* maintains cellular P balance, especially during bloom conditions that often occur at higher-than-Redfield N:P nutrient proportions^50^. We performed transcriptomic data analysis and obtained the list of genes that are potentially involved in P uptake and its regulation in *P. cordatum*. The presence of both low-affinity Pi transporter and high-affinity Pi transporter homologs means that *P. cordatum* can effectively regulate P uptake depending on the concentration of Pi in the environment. Surprisingly, we found a homolog of the PHO1 protein, which transfers Pi out of root epidermal and cortical cells into the xylem vessels in higher plants^51, 52^. This Pi exporter can also be involved in the regulation of P homeostasis in *P. cordatum.*

Dissolved organic phosphorus is the main source of P during the blooms, especially in a Pi-deficient environment. Dinoflagellates can effectively grow on various range of phosphorus-containing organic molecules, such as ATP, cytidine 5-monophosphate, fructose 6-phosphate, glucose 6-phosphate, glycerophosphate, uridine 5-monophosphate, phenylphosphate, RNA, etc.^38, 39, 53, 54^. The key enzyme allowing to release of Pi from the phosphoesters is cell-surface AP PhoA. Dinoflagellate AP protein sequences exhibit high variability and likely belong to an atypical type named PhoA^aty^^27^. Therefore, we were not able to recover *P. cordatum* AP using the protein sequence of *S. cerevisiae* AP PHO8 because of the extremely low similarity between them. So, we had to use the PhoA^aty^ sequence of haptophyte *Emiliania huxleyi* and in this attempt, we found one *P. cordatum* homolog in each transcriptome*.*

Along with well-studied APs, purple acid phosphatases (PAPs) also hydrolyze organic phosphate esters and promote phosphate acquisition in plants and microalgae. It has been observed that in the diatom *Phaeodactylum tricornutum,* PAP expression was enhanced in cells grown on organic sources of P, compared to inorganic^55^. There are 29 PAPs in *A. thaliana,* which groups into three clades by phylogeny. Only two enzymes, AtPAP11 and AtPAP12 are shown to be overexpressed under phosphate deficiency^56^. In dinoflagellates, PAP is overexpressed in P-starved *K. brevis*^31^. In this study, we found 12 putative PAP homologs in two *P. cordatum* transcriptomes that apparently play various roles in the response to P-stress. Deciphering the functions of different PAP homologs in P metabolism is a potentially perspective area for the future study.

SPX domain-containing proteins negatively regulate Pi homeostasis in plants by sensing the external and internal Pi levels^57^. Analysis of marine eukaryotic species’ transcriptomes (MMETSP dataset) revealed that SPX-related genes are widespread among different taxa of the phytoplankton, indicating their universal importance^58^. In the diatom *P. tricornutum*, two multidomain SPX-containing proteins were identified: vacuolar phosphate transporter (Vpt4), mediating vacuolar phosphate sequestration, and vacuolar transporter chaperone 4 (Vtc4)^58^. Moreover, there is one SPX-containing protein, which has the only SPX domain as a functional in *P. tricornutum.* Knockout of this gene led to significant upregulation of phosphate transporters, Vtc4, Vpt4, and an increase of alkaline phosphatase activity both in phosphate-depleted and P-repleated medium, indicating that SPX is a negative regulator of both P uptake and P-stress responses^58^. In *P. cordatum* transcriptome we found two Vtc4 homologs and one sequence having SPX as a sole functional domain, suggesting that in this species, Pi homeostasis can also be regulated in a similar way. However, this remains to be verified experimentally.

This work expands the knowledge about the regulation of life cycles in the bloom-forming dinoflagellates. We showed that P deficiency can be a factor, which induces sexual reproduction in *P. cordatum* thus affecting HAB intensity*.* The addition of phosphate-rich medium into P-deficient culture stimulated meiosis and return to the haploid vegetative stage. Analysis of transcriptomes elucidated that *P. cordatum* possesses a set of proteins that allow efficient uptake of P from various sources (inorganic and organic) in the environment with high or low phosphate concentration. The increasing frequency of harmful dinoflagellate bloom is one of the ocean challenges of the twenty-first century. We believe that an integrated approach, which includes studies of the regulation of dinoflagellate life cycles in the fluctuating aquatic environment with varying nutrient availability under different temperature and salinity regimes can contribute to the effective forecasting of dinoflagellate blooms and even help in coping with the HABs problem on a broad scale.

## Materials and methods

### Culture conditions

The culture of *P. cordatum* strain CCAP1136/16 (from The Culture Collection of Algae and Protozoa, UK) was grown in artificial seawater-based f/2 medium^59^ without added silica. Medium was sterilized by autoclaving and salinity was adjusted to 25. The vitamin mixture was sterilized by sterile filtration and added separately. The cells were grown at 18 °C under 100 µmol photons m^–2^ s^–1^ and a 12 h light:12 h dark cycle with the light period starting at 09:00 h and finishing at 21:00 h.

### Experimental design

**Figure 5:**
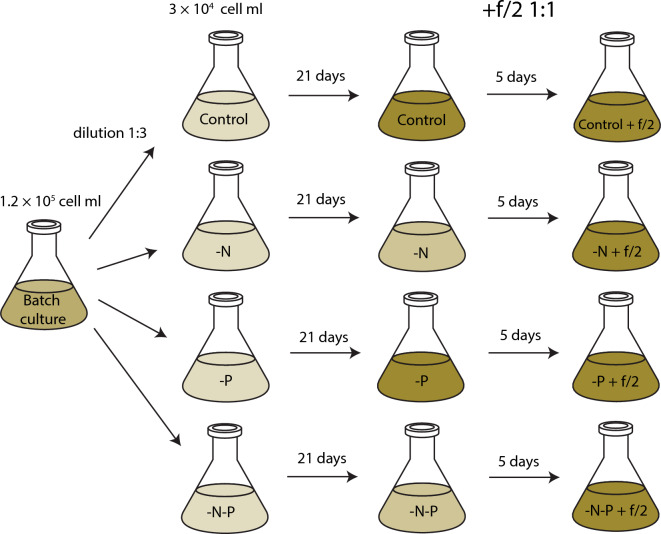
Schematic representation of the experimental design. Synchronized staring culture with a cell density of ~ 1.2 × 10^5^ cell mL was partitioned into four flasks. Each portion was diluted 1:3 with f/2 medium without added NO_3_^–^ (“-N”), PO_4_^3-^ (“-P”), or both nutrients (“-N-P”). The control culture was diluted with medium containing 880 µM NO_3_^–^ and 40 µM PO_4_^3–^. The cultures were grown for 21 days. At day 21 fresh f/2 medium containing 880 µM NO_3_^–^ and 40 µM PO_4_^3–^ was added into the cultures in proportion 1:1. The cultures were grown for 5 additional days.

The 240 mL of initial culture was grown starting from the concentration 3 × 10^4^ cell ml^–1^ in the f/2 medium containing 176 µM NO_3_^–^ and 40 µM PO_4_^3–^ for 7 days to reach a cell concentration of ~ 1.2 × 10^5^ cell ml^–1^. Then cells were transferred into complete darkness for 72 h for synchronization of the cell cycle. Afterward, the synchronized culture was partitioned into the four flasks, and each was diluted 1:3 with f/2 without added NO_3_^–^, PO_4_^3–^, or both nutrients (Fig. 5). The control culture was diluted with medium containing 880 µM NO_3_^–^ and 40 µM PO_4_^3–^. The experimental cultures were grown for 21 days. Cell density, nitrate, and phosphate concentrations were measured, and cell samples were taken for flow cytometry every three days. At day 21, fresh f/2 medium containing 880 µM NO_3_^–^ and 40 µM PO_4_^3–^ was added into the flasks in proportion to 1:1. The cultures were grown for 5 additional days; sampling was carried out on the first, third, and fifth days. The experiment was performed in triplicate.

The cell density was estimated using a Fuchs-Rosenthal counting chamber and light microscope. At least 200 cells per sample were counted. The specific growth rate was estimated as K’=$\frac{\mathrm{ln}\left(\frac{N2}{N1}\right)}{t2-t1}$ where N1 and N2 are the cell counts at times t1 and t2.

### Nitrate and phosphate concentration analysis

From each culture, a 3 mL sample was filtered through a sterile 0.22-µm pore cellulose acetate syringe filter. Phosphate and nitrate concentrations were determined spectrophotometrically according to protocols described in^60^ and^61^, respectively.

### Flow cytometry

The cell cycle of photosynthetic dinoflagellates including *P. cordatum* is regulated by the light–dark cycle. Therefore, for correctly comparing the samples, it is important to take them at a fixed time of the day. The cell samples (5–10 mL, depending on cell concentration) were taken every 3 days twice per day at 5:00 and 21:00. These time points were chosen because the maximum number of cells in the S-phase in the 12:12 light:dark period is 4 h before turning on the light, in our case it is 5:00. At 21:00 when the light is switched off, the cell division rate drops and number of cells in S-phase is close to minimum^22, 40^. The culture samples were concentrated by centrifugation (3000*g* for 3 min), fixed in 4% paraformaldehyde (PFA) at room temperature for 40 min, and rinsed in phosphate saline buffer (PBS) with 100 mM glycine for 10 min. Then the cells were transferred in 96% ethanol and kept at – 20 °C for at least 24 h for pigment extraction. Afterwards, the cells were washed thrice in PBS and incubated with 0.25 mg mL^–1^ RNase A and 0.05 mg mL^–1^ propidium iodide (Sigma-Aldrich, St. Louis, MO, USA) for minimum 30 min at room temperature. The samples were analyzed using a CytoFLEX cytometer (Beckman Coulter, Brea, CA, USA), 488 nm laser excitation, and 585/42 BP emission. At least 5,000 cells (nucleus) had been recorded for each sample. Cell cycle analysis was performed with ModFit LT software (Verity Software House, ME, USA).

### Transcriptome analysis

In order to identify the putative *P. cordatum* proteins involved in phosphate uptake, we used unannotated translated transcriptomes of two *P. cordatum* strains (CCMP1329 and CCMP2233) available in the database of the Marine Microbial Eukaryotic Transcriptome Sequencing Project (MMETSP; https://www.imicrobe.us/#/projects/104, Combined Assemblies)^62^. The query sequences were obtained from the National Center for Biotechnology Information (http://www.ncbi.nlm.nih.gov/protein) and belonged to the baker yeast *Saccharomyces cerevisiae*, the high plant *Arabidopsis thaliana* and haptophyte *Emiliania huxleyi*. The accession numbers of the queries are shown in Table 2. The search for amino-acid sequences homologous to the target proteins in *P. cordatum* transcriptomes was performed using the BLASTP algorithm in BioEdit 7.2.5 software. The BLOSUM62 scoring matrix for amino acid substitutions was chosen for the analysis. To re-verify the homology of obtained hits, the sequences with an E-value less than 10^−10^ were used as queries in the reverse BLASTP search against the nonredundant NCBI protein sequence database (https://blast.ncbi.nlm.nih.gov/Blast.cgi) and checked for the presence of functional domains using NCBI Conserved Domain Search database and PROSITE (https://prosite.expasy.org). Chosen sequences were aligned using the CllustalO algorithm and visualized by MView.

### Supplementary Information

Supplementary Figures.

## Data Availability

The translated transcriptomes of two strains of *P. cordatum* CCMP1329 and CCMP2233 analyzed in this study available from the database of the Marine Microbial Eukaryotic Transcriptome Sequencing Project (MMETSP; https://www.imicrobe.us/#/projects/104, Combined Assemblies)^62^. The query sequences were obtained from the following databases: GenBank (QHB10616.1, KAF4005909.1, QHB09608.1, KAG2518769.1, OAP03352.1, AEE74502.1, AWI47784.1, AED94948.1, AEC06729.1, AEC07951.1), RefSeq (NP_197515.1), and UniProtKB/Swiss-Prot (Q96X52, Q8GSD9) and are available from the National Center for Biotechnology Information (http://www.ncbi.nlm.nih.gov/protein).
