# Supplementary material for: Phosphorus deficiency induces sexual reproduction in the dinoflagellate Prorocentrum cordatum
Source: Sci Rep. 2023 Aug 30;13:14191. doi: 10.1038/s41598-023-41339-3 (PMC10468533; doi:10.1038/s41598-023-41339-3)

Supplementary Materials.

Phosphorus deficiency induces sexual reproduction in the dinoflagellate *Prorocentrum cordatum*.

Vera Kalinina[1]*, Mariia Berdieva[1], Nikolay Aksenov[2] and Sergei Skarlato[1]

[1] Laboratory of Cytology of Unicellular Organisms, Institute of Cytology of the Russian Academy of Sciences, St.-Petersburg, 194064, Russia

[2] Laboratory of Intracellular Membrane Dynamics, Institute of Cytology of the Russian Academy of Sciences, St. Petersburg, 194064, Russia

* Correspondence: Vera Kalinina, E-mail: verakamakalinina@gmail.com

Suppl. Fig.1. Examples of raw flow cytometry data. Flow histograms showing DNA intensity of the samples taken at the time points 6 days and 18 days at 5:00 **(a)** and 21:00 **(b)** in the control and P-deficient cultures.

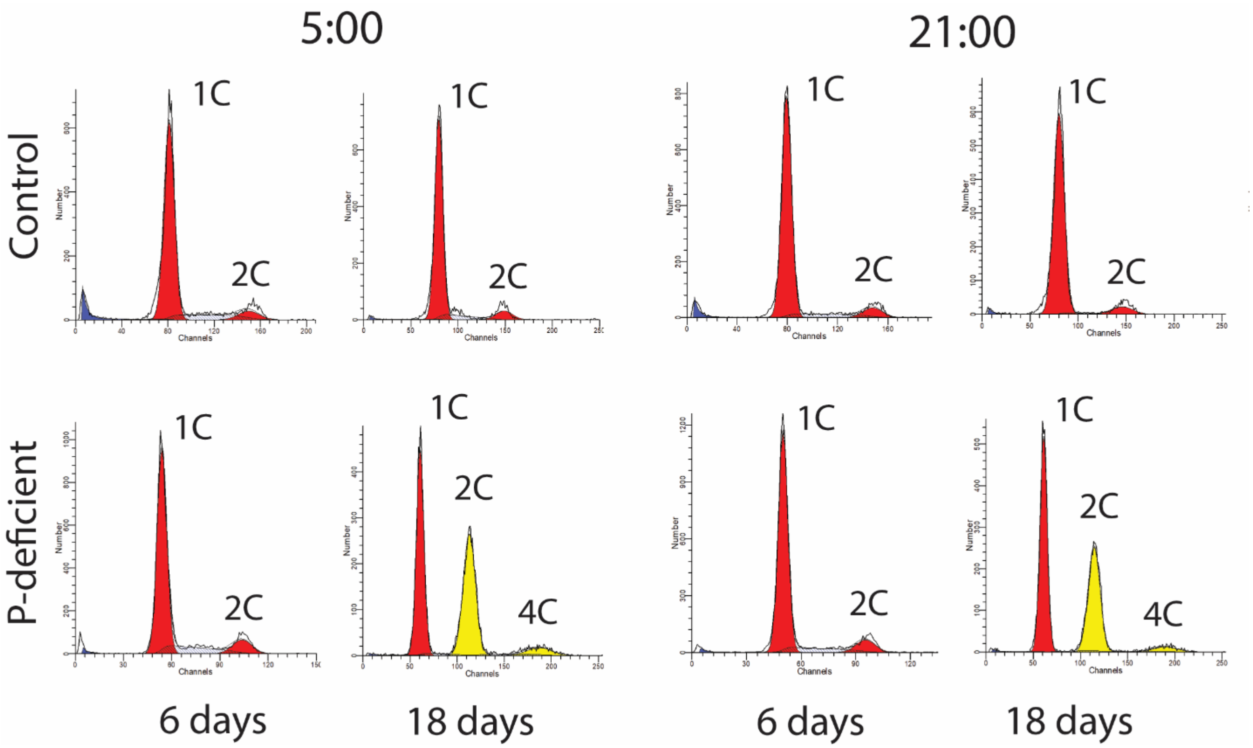

Suppl. Fig. 2. Percentage of cells in the different phases of the cell cycle during 21-day experimental period. Shown are means from the triplicated experiments in the control and P-deficient cultures.

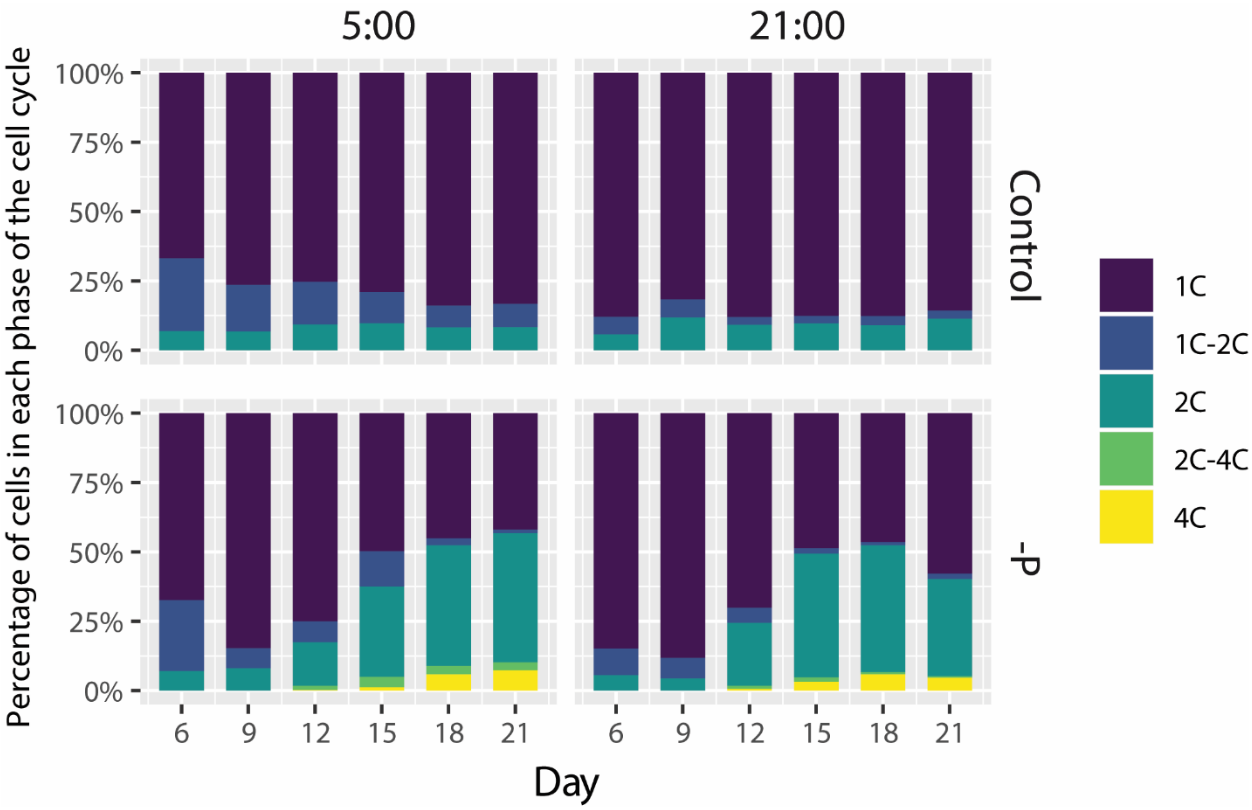

Suppl. Fig. 3. Multiple sequence alignment of *P. cordatum* PHO84 homologs with homologous sequences from various species. The sequences used in the alignment are: *Saccharomyces cerevisiae* (Sc_Pho84, GenBank ID: QHB10616.1), *Pholiota nameko* (Pn, UniProtKB ID: Q96X52), *Arabidopsis thaliana* (At, GenBank ID: AED94948.1), *Oryza sativa* (Os, UniProtKB ID: Q8GSD9). Conserved sequences of proton-coupled transporters generated by the motif-building program MEME are black-boxed. The glycine-rich phosphate binding motif GXGXGG shared by proton-coupled phosphate transporters in plants, fungi, bacteria, and mammals shown in the red box. Sites corresponding to *S. cerevisiae* Arg168, Asp358 and Lys492 are shown in purple boxes and marked with arrows.

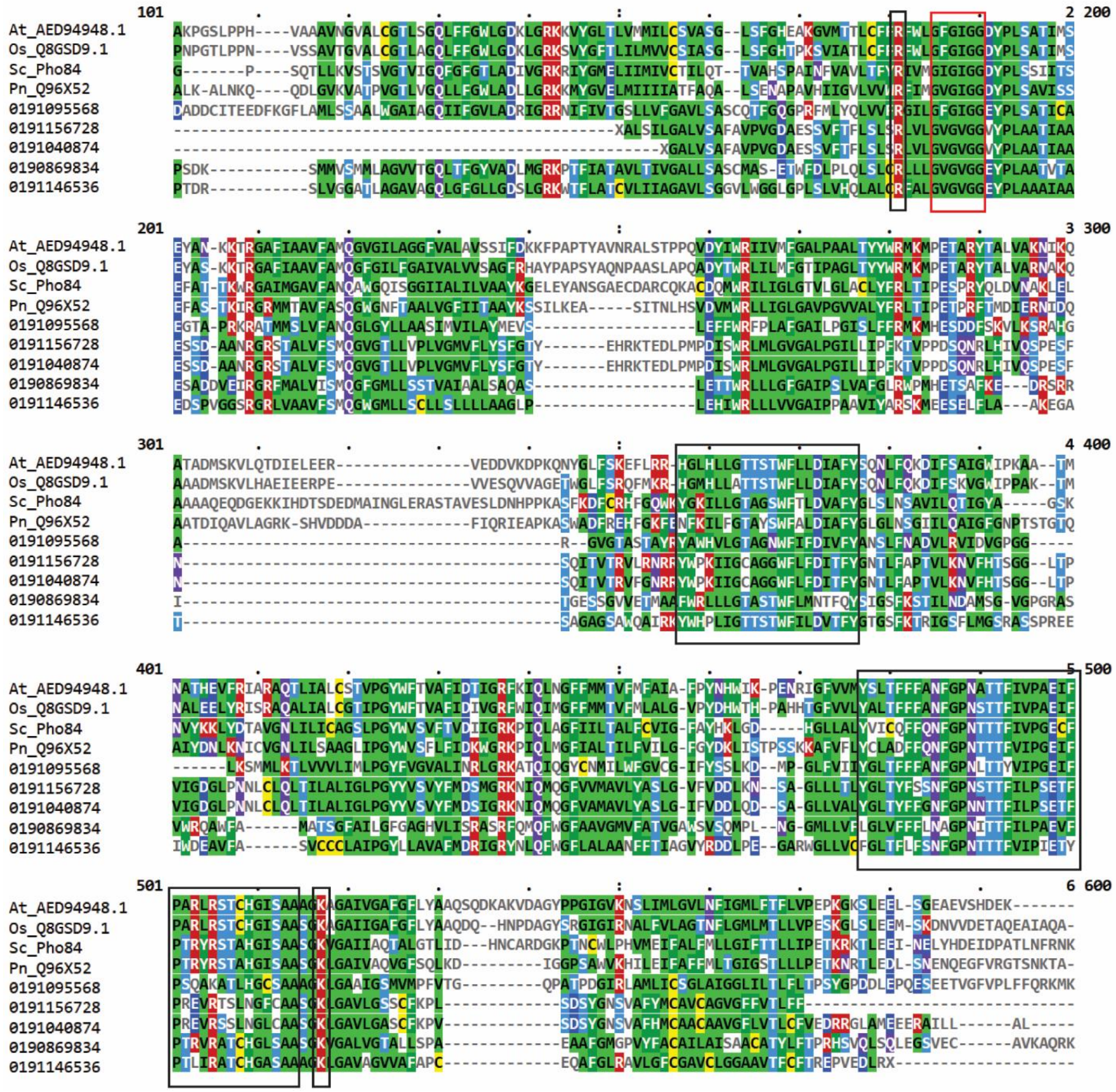
101
200
201
300
301
400
401
500
501
600
At_AED94948.1
Os_Q8GSD9.1
Sc_Pho84
Pn_Q96X52
0191095568
0191156728
0191040874
0190869834
0191146536

Suppl. Fig. 4. Multiple sequence alignment of *P. cordatum* PAPs with homologous sequences of *A. thaliana* PAP11 (GenBank ID: AEC06729.1) and PAP12 (GenBank ID: AEC07951.1). The conserved sequence motifs of PAPs identified by Schenk et al.[36] are boxed.

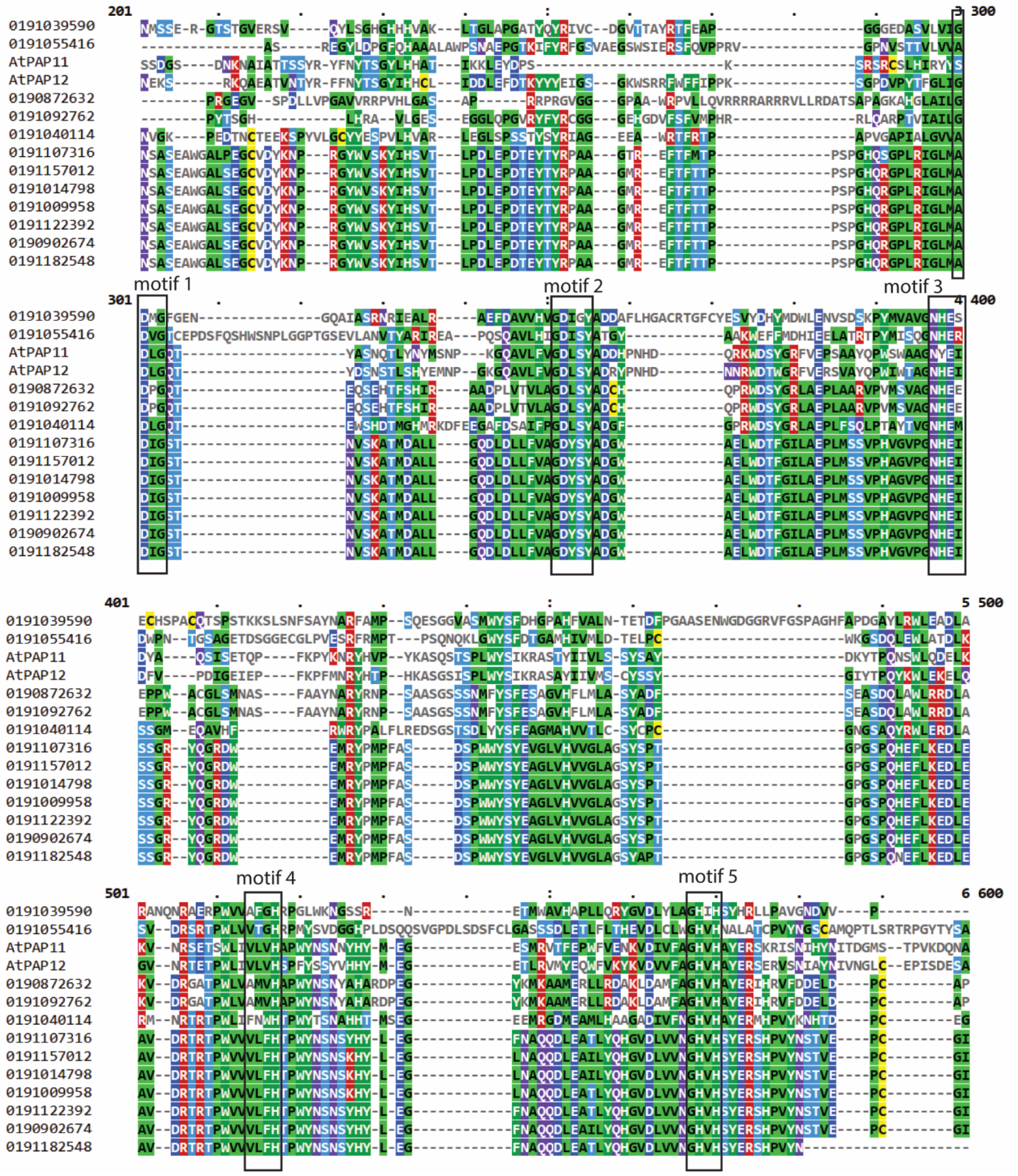

Supplement: Supplementary file 1 — Supplementary Figures. [file 41598_2023_41339_MOESM1_ESM.pdf]
